# Phenotypic Variation across Chromosomal Hybrid Zones of the Common Shrew (*Sorex araneus*) Indicates Reduced Gene Flow

**DOI:** 10.1371/journal.pone.0067455

**Published:** 2013-07-10

**Authors:** P. David Polly, Andrei V. Polyakov, Vadim B. Ilyashenko, Sergei S. Onischenko, Thomas A. White, Nikolay A. Shchipanov, Nina S. Bulatova, Svetlana V. Pavlova, Pavel M. Borodin, Jeremy B. Searle

**Affiliations:** 1 Departments of Geological Sciences and Biology, Indiana University, Bloomington, Indiana, United States of America; 2 Institute of Cytology and Genetics, Siberian Branch of Russian Academy of Sciences, Novosibirsk, Russia; 3 Kemerovo State University, Department of Zoology and Ecology, Kemerovo, Russia; 4 Department of Ecology and Evolutionary Biology, Cornell University, Ithaca, New York, United States of America; 5 Computational and Molecular Population Genetics (CMPG) Lab, Institute of Ecology and Evolution, University of Bern, Bern, Switzerland; 6 A.N. Severtsov Institute of Ecology and Evolution, Russian Academy of Sciences, Moscow, Russia; 7 Department of Biology, University of York, York, United Kingdom; Australian Museum, Australia

## Abstract

*Sorex araneus*, the Common shrew, is a species with more than 70 karyotypic races, many of which form parapatric hybrid zones, making it a model for studying chromosomal speciation. Hybrids between races have reduced fitness, but microsatellite markers have demonstrated considerable gene flow between them, calling into question whether the chromosomal barriers actually do contribute to genetic divergence. We studied phenotypic clines across two hybrid zones with especially complex heterozygotes. Hybrids between the Novosibirsk and Tomsk races produce chains of nine and three chromosomes at meiosis, and hybrids between the Moscow and Seliger races produce chains of eleven. Our goal was to determine whether phenotypes show evidence of reduced gene flow at hybrid zones. We used maximum likelihood to fit *tanh* cline models to geometric shape data and found that phenotypic clines in skulls and mandibles across these zones had similar centers and widths as chromosomal clines. The amount of phenotypic differentiation across the zones is greater than expected if it were dissipating due to unrestricted gene flow given the amount of time since contact, but it is less than expected to have accumulated from drift during allopatric separation in glacial refugia. Only if heritability is very low, *N_e_* very high, and the time spent in allopatry very short, will the differences we observe be large enough to match the expectation of drift. Our results therefore suggest that phenotypic differentiation has been lost through gene flow since post-glacial secondary contact, but not as quickly as would be expected if there was free gene flow across the hybrid zones. The chromosomal tension zones are confirmed to be partial barriers that prevent differentiated races from becoming phenotypically homogenous.

## Introduction

The Common shrew, *Sorex araneus*, provides an unparalleled opportunity to study the process of speciation. Robertsonian karyotypic variation, in which chromosomal arms are rearranged at the centromeres, subdivides this species into more than 70 karyotypic races [1], [2]. Members of a race by definition share the same combination of acrocentric and metacentric chromosomes in a geographically contiguous part of the parent species' range [3].

**Figure 1:**
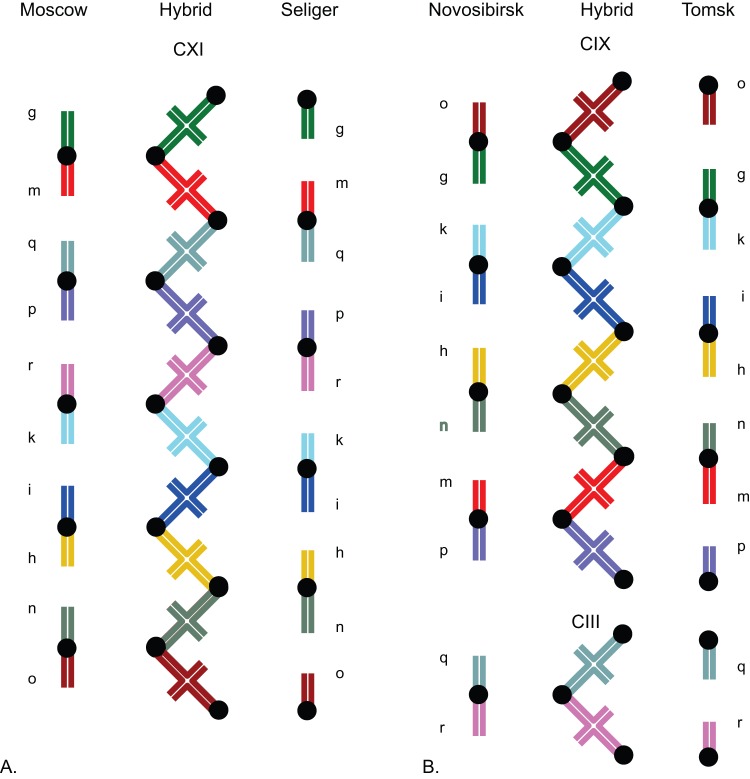
Karyotypes of *Sorex araneus.* Metacentric chromosomes of pure race and hybrid individuals at the Moscow-Seliger hybrid zone (A) and the Novosibirsk-Tomsk zone (B). Homologous arms are labeled and color coded.

Where parapatric karyotypic races make contact, Robertsonian incompatibilities cause them to form tension hybrid zones. *Sorex araneus* possesses eighteen fundamental arms, excluding the sex chromosomes, which are labeled alphabetically from largest to smallest (*a-u*); arms *g-r* vary in how they are combined into chromosomes [4], [5]. Hybrid incompatibilities arise when homologous fundamental arms are combined into different metacentric chromosomes in the two parent races. The mismatches cause heterozygote chromosomes to align in chains and rings of varying complexity during the first division of meiosis (**Figure 1**). In simple heterozygotes, two acrocentric arms from one race align with a single metacentric from the other (a chain of three, CIII); in complex heterozygotes, several metacentrics align in longer chains or rings. For complex heterozygotes, problems in pairing at prophase and difficulty in segregation at anaphase result in reduced hybrid reproductive fitness. The role of meiotic drive in fixing metacentrics within races and effects of metacentric incompatibilities between races have been studied in detail at several hybrid zones [5]–[13]. Simple heterozygotes cause only marginal reductions in fitness, but hybrids with large rings or long chains can be substantially unfit [7], [14]–[17]. The lowered fitness maintains the sharp hybrid zones by preventing the metacentrics from introgressing between races.

Hybrid unfitness can, in principle, lead to speciation. In the classical chromosomal model of speciation, karyotypic incompatibility leading to reduced fertility of hybrids was considered as the first stage in reproductive isolation [18], [19]. Modern versions of the chromosome speciation model suggest that the reduction in gene flow is concentrated in the genes located on the rearranged chromosomes because of cross-over suppression [20]–[22], an effect heightened close to the chromosomal breakpoint [23]–[25]. Especially when combined with reinforcement, the reduction in gene flow can result in genetic and phenotypic differentiation, reproductive isolation, and ultimately speciation [22], [26].

Intriguingly, however, substantial gene flow occurs across *S. araneus* hybrid zones despite reduced hybrid fitness. Genetic differentiation between races is weak, as indicated by allozyme markers [27], [28], mitochondrial DNA [8], [9], and microsatellite markers [29], even at zones where chains of nine (CIX) or eleven chromosomes (CXI) are formed in heterozygotes. Indeed, F-statistics on multilocus microsatellite markers suggests that genetic differences between populations within races are higher than the differences between races [29]. Some studies have even found that fertility may be relatively high for some types of complex heterozygotes and that metapopulation dynamics can overwhelm the weak barrier effects of reduced hybrid fitness [15], [16], [30]. The lack of genetic differentiation between races contrasts with the high level of differentiation between sister species [31], [32]. The persistence of gene flow has led some researchers to conclude that Robertsonian rearrangements do not promote speciation in *S. araneus*, but are instead merely remnants of past allopatric differentiation being lost following secondary contact [28], [29], [33].

In this paper we look at clines of morphological variation across two well-studied hybrid zones to determine whether phenotypic differentiation between hybridizing races is sharply delineated in similar fashion to the metacentrics or weakly defined like the genetic markers. We measured clines in skull and mandible shape using geometric morphometrics across the Moscow-Seliger (M-S) hybrid zone in European Russia [10], [34] and the Novosibirsk-Tomsk (N-T) zone in Siberia [11], [35], [36]. Heterozygote offspring from the two zones produce especially long meiotic chains of eleven and nine and three chromosomes respectively (**Figure 1**). We chose to look at skull and mandible shape because these trait complexes are multivariate, polygenic (including autosomal genes), and easily measured. In mice, for example, there are as many as 50 mandible QTLs scattered over all 19 autosomes [37]–[40]. Clines in these phenotypic traits should therefore indicate gross differentiation in the autosomal genome. The disadvantage of skeletal traits as measures of genetic differentiation is that an unknown component of their variance is environmental, though studies in other mammalian taxa suggest that overall heritability in these multivariate phenotypic systems is likely to fall between 0.3 and 0.5 [41], [42]. Ambiguities of interpretation related to heritability are discussed below.

This study is the first to systematically examine phenotypic clines across *S. araneus* hybrid zones. Previous morphometric studies have looked at morphometric variation in pure and hybrid race individuals at selected hybrid zones [43]–[47], others have looked at phenotypic variation across large segments of the species' range [48]–[50], and a few have looked at morphometric differences between hybridizing sister species of the *S. araneus* group [51]–[53]. While phenotypic clines have never been studied directly in *S. araneus*, these previous studies suggest that phenotypic differentiation between races is often small but statistically significant, that differentiation at the population level is often greater than differentiation between races, as with genetic markers, and that phenotypic differentiation between groups of races can be as large as between sister species.

Our specific aims are to determine: (1) whether phenotypic clines exist across these karyotypic hybrid zones; (2) whether the centers and widths of the phenotypic clines coincided with the centers and widths of chromosomal clines; (3) whether phenotypic differentiation is greater than differentiation in genetic markers; and (4) whether phenotypic differentiation is greater than expected if there was substantial gene flow.

## Materials and Methods

### Samples

**Figure 2:**
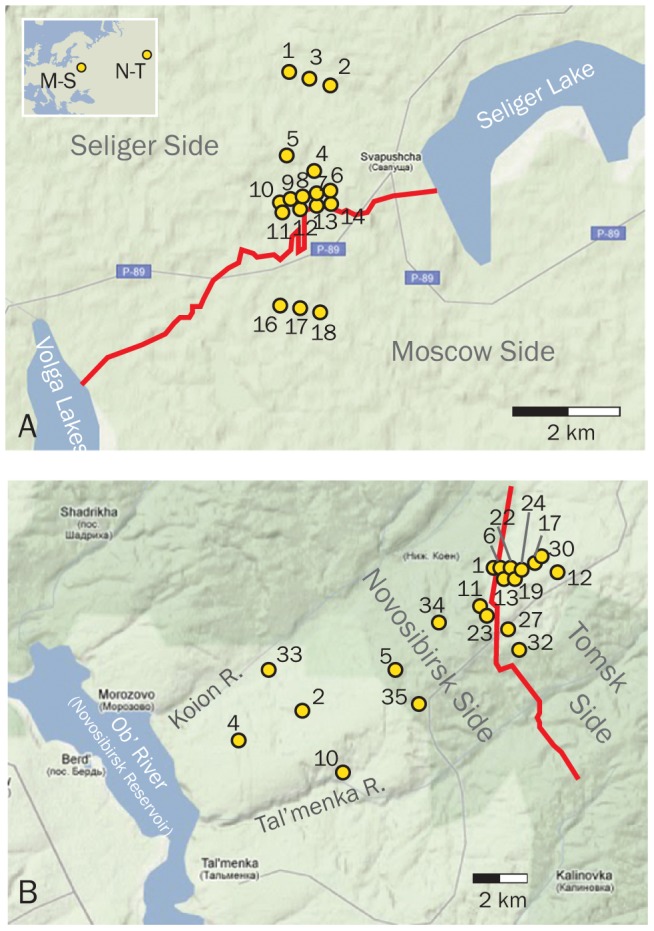
Maps of the Moscow-Seliger and Novosibirsk-Tomsk hybrid zones. Maps showing the sublocalities where the Moscow-Seliger clines (A) and Novosibirsk-Tomsk clines (B) were sampled. See **Table S1** for details of the numbered sites. Background maps courtesy of Google Maps^TM^. Centers of the two chromosomal hybrid zones are shown in red.

Shrew skulls were photographed for morphometric analysis. The specimens represent two *Sorex araneus* hybrid zones that were the subject of previous chromosomal studies: (1) the Moscow-Seliger (M-S) zone in European Russia, where individuals were allocated to 18 sublocalities derived from five parallel trap lines crossing the hybrid zone (**Figure 2A** **)**; and (2) the Novosibirsk-Tomsk (N-T) hybrid zone in Siberia, where individuals were collected from 20 sublocalities scattered on both sides of the metacentric hybrid zone center (**Figure 2B**) and two additional pure-race localities farther removed from the hybrid zone (see Table S1 for locality details for both hybrid zones). The chromosomal clines at these two hybrid zones were recently published by some of us [10], [11], [34]; the methods used to extract chromosomes and to estimate clines are described in full there. Briefly, however, karyotypes for each individual were determined using G-banding procedures [54]. Karyotypic clines were fit for each distinct metacentric chromosome using a maximum likelihood fit in two geographic dimensions and the chromosomal cline centers and widths estimated from those [55]. Trapping, handling and euthanasia of animals followed protocols approved by the Animal Care and Use Committees of the A.N. Severtsov Institute of Ecology and Evolution and the Institute of Cytology and Genetics of the Russian Academy of Sciences. No additional permits are required for research on non-listed species in Russia. Specimens were deposited the research collections of the Department of Zoology and Ecology, Kemerovo State University, Kemerovo, Russia. Skulls and mandibles were photographed at KSU by two of us (VBI, SSO).

### Shape and size

A total of 282 specimens were used for morphometric analysis. These include 152 individuals from the M-S Zone (M = 50; S = 73; Hybrid = 29) and 130 from the N-T zone (N = 61; T = 42; Hybrid = 27). These specimens were grouped by sublocality for cline fitting. Fewer sublocalities were used in this study than in the chromosomal analyses [10], [11] because broken specimens and small samples made some of those sublocalities untenable for morphometric analysis. The sublocalities used in this study are described above, shown in **Figure 2**, and described in **Table S1**.

**Figure 3:**
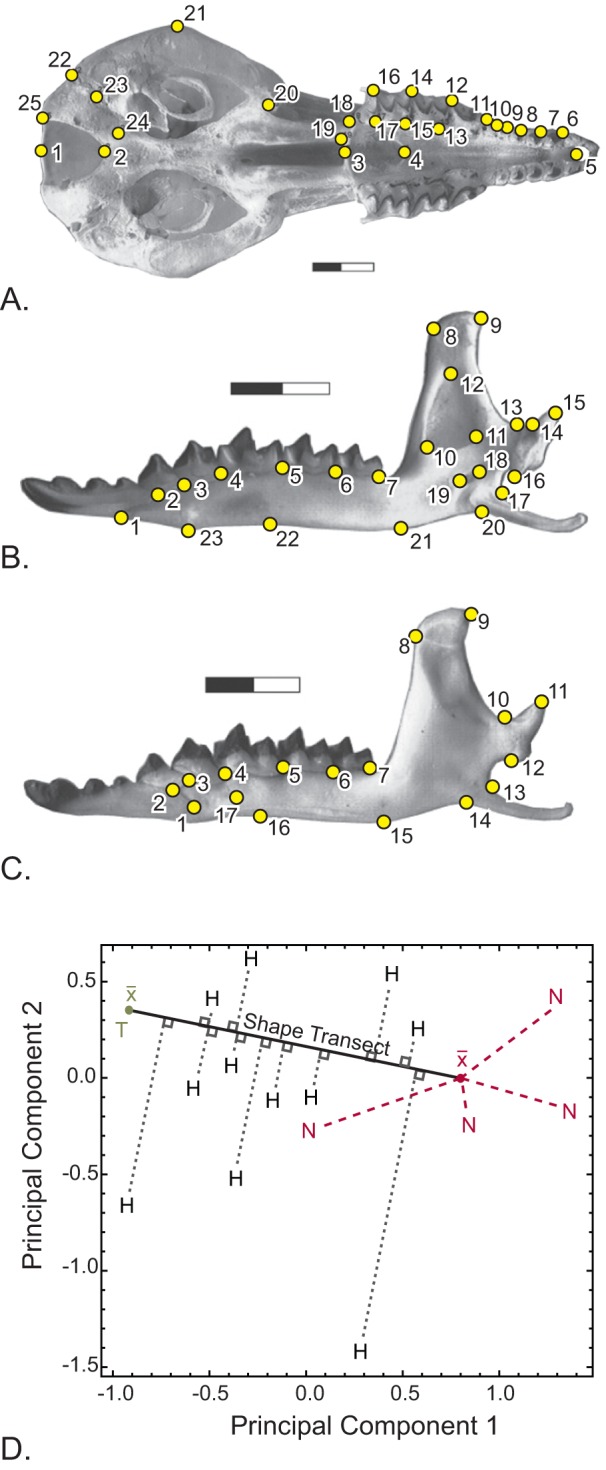
Landmark scheme. Landmarks of the ventral cranium (A), medial mandible (B), and lateral mandible (C). **D.** Example (Novosibirsk-Tomsk hybrid zone) showing graphically how the shape transect is calculated. A line is drawn through multivariate shape space between the two means of the pure race localities and the means of the hybrid localities are projected onto it. The result is a univariate axis that best describes the gradient in shape from one side of the hybrid zone to the other. For simplicity, the projections of the hybrids in this example all project between the means of the two races, but in reality they may also project outside them.

Each cranium was digitally photographed in ventral view and each mandible in both medial and lateral views. Two-dimensional Cartesian coordinates were recorded for biologically homologous landmarks on each structure (**Figure 3** **, Table S2**). Note that all of the landmarks on the lateral mandible are equivalent to landmarks on the medial mandible, except for landmark 17 (mental foramen). Those two datasets are therefore expected to be highly correlated. Shape and size data were collected with tpsDig by FJ Rohlf. The landmarks and associated data are available from Indiana University ScholarWorks (http://hdl.handle.net/2022/15279).

Landmarks were aligned using generalized Procrustes analysis (GPA) to remove differences in size, translation and rotation. The aligned shapes were projected orthogonally into tangent space for further analysis [56]. The mean was subtracted from the GPA superimposed landmarks to center their coordinate system, the covariance matrix of the residuals was calculated, and the residuals were projected onto the eigenvectors of the covariance matrix to obtain principal component scores that could be used as shape variables for subsequent analysis [57]. Note that all of the shape variance found in the original landmark data is preserved in the complete set of principal component scores.

Differences in shape between individuals or populations were measured as Procrustes distance, *D*, which can be calculated either as the square-root of the sum of the squared differences between corresponding landmarks after they have been superimposed, or as the square-root of the sum of the squared differences between PC scores for all principal components. *D* is used in several of our analyses, notably the estimation of phenotypic clines (see below). Thin-plate spline deformation grids were used to illustrate differences in shape [57]. Size of each element was measured as centroid size, which is the square-root of the sum of the squared distances between each landmark and the object's centroid [58]. Analyses were performed with the Morphometrics for Mathematica 9.0 add-in for Mathematica™ (http://hdl.handle.net/2022/14613) [59].

### Tests for significant difference in shape

We measured phenotypic differentiation among several combinations of samples, as explained below. MANOVA was used to test the statistical significance of differences in mean shape between samples, notably among the pure race and hybrid groups at each of the two hybrid zones.

### Q_ST_ and differences expected from drift

*Q_ST_* the analogue of *F_ST_* for population differentiation in quantitative phenotypic traits, was used to measure differentiation between pure race samples on either side of both hybrid zones:
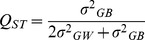
(1)where 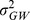 is the additive genetic variance within samples and 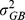 is the genetic variance among samples [60]–[62]. An unbiased estimate of *Q_ST_* and its standard error were estimated using the jack-knife procedure of Weir and Cockerham [63]. Since the additive genetic variance of our populations was unknown, we substituted the phenotypic variances, 0.5 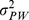 and 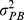, which assumes that that heritability (*h^2^*) within samples is 0.5 and that environmental variance among samples is randomly distributed or absent [41]. If this heritability is an overestimate, then our *Q_ST_* values will have been underestimated [64]. The ambiguities of interpretation that arise from uncertainty about heritability are discussed below. Analyses were performed with Mathematica ™ 9.0.

### Estimation of selection on the phenotypes

The proportion of phenotypic differentiation to neutral genetic differentiation was measured with the ratio *Q_ST_/F_ST_*, where *F_ST_* a similar parameter used for differentiation in alleles or frequencies of genetic markers [60], [62]. If *F_ST_* is estimated from neutral markers, then the ratio provides a measure of whether phenotypic differentiation is greater than expected due to drift [61]: if *Q_ST_*/*F_ST_* >1, then the phenotypes may be (or have been) under differentiating selection in the two populations; if *Q_ST_/F_ST_* equals 1 then the phenotypes may have differentiated by drift; or if *Q_ST_/F_ST_* <1 then the phenotypes may be under stabilizing selection. *F_ST_* values for these two hybrid zones were published by Horn et al. [29] based on 16 microsatellite loci. These estimates were based on the same populations as our morphometric results. These estimates were supplemented by another published *F_ST_* estimates for the Moscow-Seliger zone at a different locality (11 autosomal microsatellite loci [65]).

Drift versus selection was also assessed by comparing the observed variance between populations to the amount expected from random drift:
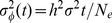
(2)Where *σ^2^_φ_(t)* is the expected variance of phenotypic change due to drift at time *t*, *h^2^* is the heritability, *σ^2^* is the phenotypic variance of the trait, *t* is the number of generations elapsed, and *N_e_* is the effective population size [66], [67]. As with *Q_ST_* heritability was assumed to be 0.5; if the true heritability is higher, then the amount of differentiation due to drift will be greater, whereas if it were lower (which it probably is) the amount of differentiation due to drift will be smaller. *N_e_* was unknown in our populations so we used two estimates, a conservatively small one derived from field censuses of local populations of *S. araneus* where *N_e_* = 70 [68] and a conservatively large one estimated from diversity in molecular markers where *N_e_* = 70,000 [69]. The true value undoubtedly lies between these two extreme estimates, probably closer to the small one, so the two estimates we derive from Equation 2 should confidently bracket the plausible range of phenotypic differentiation due to drift. The range of interpretation arising from uncertainty about heritability and population size are discussed below. Analyses were performed with Mathematica ™ 9.0.

### Hybrid zone widths

Hybrid zone widths were estimated by fitting a *tanh* cline model to the phenotypic data at the M-S and N-T hybrid zones. The *tanh* model [70]–[72] is equivalent to the logistic regression model of Gay et al. [73] when the two tails of the cline have the same slope. We used the *tanh* model because the logistic model can only handle values between 0 and 1, whereas our data, which have 0 and 1 centered on the means of the two opposing populations, have data points that are smaller than 0 and larger than 1. The phenotypic mean of each sublocality (see **Figure 2**) was calculated and a one dimensional *tanh* model was fit to those means.

*Tanh* models are normally fit to chromosomal data taken from a transect across a hybrid zone between end-point populations on either side where the proportion of a particular metacentric is 1 at the end where it is fixed in the population and grades to 0 on the other side where the metacentric is absent. We standardized our phenotypic data to vary in the same way by calculating the mean of pure race localities on either side of the zone and standardizing the phenotypic variations so that those means had values of 0 and 1.

To do this, we first reduced the multivariate shape data to a single descriptive variable that describes the phenotypic differences between the two races. First a line in multivariate shape space that passes through the mean shapes of the two pure race samples was calculated. The means of each locality were projected onto that line, providing a summary of phenotypic variation with respect to the two pure races for all of the localities (**Figure 3d**). Sublocalities with fewer than four individuals were not used. This method of reducing dimensionality is an extension of a simpler approach used by Gay et al. [73], who used the first principal component (PC1) of variation as a univariate summary of multivariate phenotypic variation. Using PC1 to measure a cline has the disadvantage that the first principal component summarizes most of the variance in the dataset, but it does not necessarily describe the differences between the two groups (e.g., it may describe sex-related variation, variation in size or age, or simply variance in random outliers). Our approach uses the major axis that best distinguishes the two races, which may or may not be parallel to PC1. The geographic axis of the *tanh* fitting was calculated as the distance of each sublocality to its nearest neighbor point on the metacentric cline center (in kilometers). Distances to localities on the Novosibirsk and Seliger sides of the two zones were arbitrarily given negative numbers for purposes of plotting data and fitting cline curves. We then standardized the shape variable so that the mean of the pure race localities on the ‘negative’ side of the cline (Novosibirsk and Seliger races) was 0 and the mean on the positive side (Tomsk and Moscow races) was 1. To do this we subtracted the Novosibirsk (or Seliger) mean from the variable and divided by the phenotypic distances (*D*) between the two pure race locality means.

The width (*w*) and center (*c*) of the hybrid zone were then estimated for each morphological element using maximum likelihood to fit a *tanh* curve:
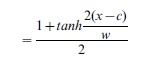
(3)where *y* is the standardized phenotypic distance expected under the model, *x* is the position of the sampling locality as described above, *c* is the center of the phenotypic cline being estimated, and *w* is the width of the phenotypic cline [70]–[72].

Standard errors for the cline parameters were estimated with bootstrapping [74]. For each of 1000 iterations, each sublocality was resampled with replacement and its mean recalculated after Procrustes superimposition. Those means were resuperimposed, the morphological transect between the races was reestimated, and the cline was refit to generate a distribution for *w* and *c*. Standard errors were calculated as one standard deviation of the resampled parameters on either side of the median (i.e., plus and minus the 34.1th percentile). The distributions of these parameters are skewed so the positive and negative standard errors are not equal. These standard errors take into account uncertainties associated with the sampling at each sublocality, with the Procrustes superimpositions, with the estimations of the race means, with the shape transect between the race means, and with the fitting of the *tanh* cline. Standard errors cannot be estimated from the likelihood function itself because of the Procrustes superimposition, which is an iterative best-fit algorithm that is different for every resampling. Analyses were performed with Mathematica ™ 9.0.

### Estimations of the minimum age of the hybrid zones

When two allopatric populations first come into secondary contact, a sharp, narrow cline forms. If there is gene flow between the populations, then the cline grows wider as the phenotypes, genotypes, or karyotypes introgress into the adjacent populations [75]. The minimum time since secondary contact can be estimated based on the time it would have taken the cline to have grown to its present width if there was no barrier to gene flow [75]:
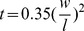
(4)where *t* is time in generations, *w* is the present width of the cline, and *l* is the root-mean-square gene flow distance. Generation time in shrews is approximately 1 year. Gene flow is measured as the average dispersal distance of individual shrews per generation, which we estimated at 1 km following Polyakov et al. [11] (see discussion below). The time since secondary contact will, of course, have been much longer than predicted by this equation if barriers to gene flow prevent dissipation of the cline. Analyses were performed with Mathematica ™ 9.0.

## Results

### Differentiation in shape and size between hybridizing races

Shape differentiation between pure races was approximately the same at the two hybrid zones (**Table 1**). *Q_ST_* values ranged from 0.012 to 0.040, and the differences in mean shape between the races were statistically significant for all three data sets at both hybrid zones when tested with MANOVA.

**Table 1:** Phenotypic differentiation at hybrid zones.

	Q*_ST_*	F	P
	*Novosibirsk-Tomsk Zone*		
	*Shape*		
*Skulls*	0.040±0.002	1.3×1063	0.00
*Medial Mandible*	0.017±0.001	1.3×1056	0.00
*Lateral Mandible*	0.012±0.001	3.96	0.00

	*Size*		
*Skulls*	0.084±0.007	5.24	0.02
*Medial Mandible*	0.132±0.008	0.96	0.00
*Lateral Mandible*	0.225±0.009	22.04	0.00

	*Moscow-Seliger Zone*		
	*Shape*		
*Skulls*	0.021±0.002	1.95	0.01
*Medial Mandible*	0.017±0.001	7.65	0.00
*Lateral Mandible*	0.012±0.001	2.93	0.00

	*Size*		
*Skulls*	0.013±0.001	1.44	0.23
*Medial Mandible*	0.000±0.001	0.01	0.97
*Lateral Mandible*	0.002±0.001	0.27	0.61

*Q_ST_* is the differentiation in quantitative traits between the pure races on either side of the zone. Results of MANOVA tests for differences among the pure races and/or hybrids are reported as *F* (the F-ratio statistic) and *P* (the probability that the observed differences are due to chance).

Size differentiation of the elements was high at the N-T zone, but nearly absent at the M-S zone. *Q_ST_* for size ranged from 0.084–0.225 at the N-T zone and from 0.000–0.013 at the M-S zone. The large size difference between the N-T races is consistent with a previous size-based morphometric analysis that found that Novosibirsk shrews were significantly smaller than Tomsk shrews [45].

**Figure 4:**
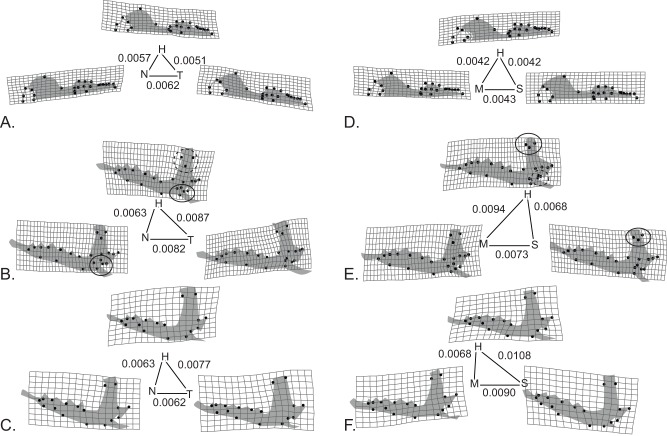
Comparison of pure race and hybrid phenotypes. (**A–C**) skulls, medial mandibles and lateral mandibles from the Novosibirsk-Tomsk hybrid zone. (**D–F**) the same three structures from the Moscow-Seliger hybrid zone. The mean shape of each hybrid and pure race groups is shown as a thin-plate spline deformation from the grand sample mean (exaggerated by 10x). Cartoon outlines of each structure have been added to make the landmark configurations more intelligible. Solid circles highlight examples where the hybrid phenotype is like one of the parents; dashed circles highlight unique features of the hybrids. Distances between the hybrid and pure race means are reported in Procrustes units along edges of a triangle proportional to those distances.

The hybrid phenotypes were not directly intermediate in shape between those of the two parent races at either hybrid zone. Differences in the mean shapes of the pure race samples and hybrids are shown in **Figure 4**. In this figure, the morphological distance of the hybrid samples to the parents is represented by the lengths of the sides of the triangles; if the hybrid phenotype were precisely intermediate between the parent races, then the hybrids would lie on the line connecting the parents. The finding that the hybrids are not intermediate, which can result from overdominance, epistasis, or other non-additive effects, is common in multivariate polygenic traits [38]. Note that individuals with pure race karyotypes could be of hybrid origin if an F1 hybrid backcrossed with an individual of one of the parent races. Genetic recombination in the hybrid parent could cause the apparently pure race offspring to more resemble the hybrid phenotype. If back crossing occurred asymmetrically between the parent races it could contribute to the hybrid phenotypes being more similar to one than the other.

### Phenotypic clines

**Figure 5:**
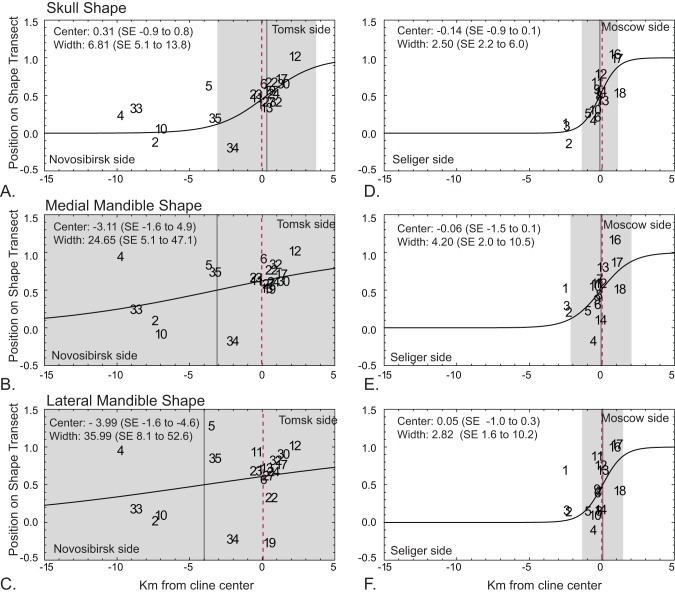
Phenotypic clines across the two hybrid zones. **A–C.** Novosibirsk-Tomsk hybrid zone. **D–E.** Moscow-Seliger hybrid zone. Horizontal axes show the distance in km from the center of the metacentric hybrid zone (Novosibirsk and Seliger distances shown as negatives) and vertical axes show the shape transects between the pure race samples (standardized with Novosibirsk and Seliger means equal to 0 and Tomsk and Moscow equal to 1). The center of the metacentric zone is highlighted with a dashed red line. The ML estimate of the phenotypic cline is shown in black, with its center marked by a vertical grey line and its width indicated by light grey shading. Data points are labeled using the locality numbering system in **Figure 2** and **Table S1.**

The phenotypic clines were substantially wider at the N-T zone (6.8 to 36.0 km) than at the M-S zone (2.5 to 4.2 km) (**Figure 5**). The widths of the phenotypic clines parallel those of the metacentrics at these two zones. The cline widths at the N-T zone of the metacentrics forming the long chains (CIX) are 8.5 km (CI = 6.2 to 12.8 km) and the short chains (CIII) are 52.8 km (CI = 22.6 to 199.2 km) [11] and the metacentrics forming the long chain (CXI) have a width of 3.3 km (CI = 2.7–4.5 km) at the M-S zone [10], [34]. The wide clines in mandible shape at N-T were more linear than sigmoidal. The geographic centers of the phenotypic clines were very close to the centers of the metacentric clines, all within 0.3 km except for mandible shape at N-T, which were offset by 3 to 4 km (**Figure 5**).

No clines were found in any of the size data sets except at the N-T zone where the medial mandible had a cline 1.7 km wide centered 0.58 km toward the Tomsk side of the metacentric zone center and the lateral mandible had a cline that was 0.48 km wide and centered 1.58 km toward the Tomsk side of the zone. These sharp clines in mandible size are consistent with the distinctly larger size of the Tomsk race individuals [45].

### Expectation of differentiation at the hybrid zones due to drift

We found that the amount of differentiation between the hybridizing races is smaller than expected from drift, though this interpretation depends on assumptions about effective heritability, population size, and the interval of time that drift operated. Those assumptions are relaxed below. We estimated the amount of differentiation expected from drift during the pre-contact period of allopatry and compared it to the observed differences at the two hybrid zones. If gene flow is substantially blocked between the races, we expect them to differ at least by the amount of drift that would have accumulated during their period of allopatry, not to mention subsequently. However, the observed differences across the two hybrid zones (0.18 to 0.32 standard deviates) were smaller than expected from drift (0.3 to 8.5 standard deviates) (**Table 2**). This finding seems to suggest that phenotypic differentiation between the races has been lost through gene flow, but it is based on assumptions about heritability, effective population size (*N_e_*), and the amount of time available for drift to accumulate (**Equation 2**). We discuss each of these in turn here and consider alternative scenarios.

**Table 2:** Estimates related to the age of the hybrid zone.

	Observed Differences		Years since secondary contact
	(Procrustes units)	(SD Units)	
*Novosibirsk-Tomsk Zone*			
Skulls	0.007	0.32	5.7
Medial Mandibles	0.010	0.22	74.4
Latateral Mandibles	0.008	0.19	158.7

*Moscow-Seliger Zone*			
Skulls	0.004	0.20	0.8
Medial Mandibles	0.007	0.18	2.2
Lateral Mandibles	0.008	0.22	1.0

The first two columns report the observed differences in Procrustes units and standard deviates and the last column reports the number of years since secondary contact if there is no barrier to gene flow. The amount of differentiation expected by drift over 10,000 years (see text) is 8.5 standard deviates if small effective population size is assumed (*N_e_* = 70) and 0.3 standard deviates if a large population is assumed (*N_e_* = 70,000).

The rate of drift depends on the heritability of the traits. Our estimates arbitrarily use a heritability of *h^2^* = 0.5. This is probably the highest plausible estimate based on work on heritability of skull and mandible shape in other species [41], [42]. If heritability is lower, then the amount of divergence expected from drift will also be proportionally lower. If heritability was at the small end of the plausible range (e.g., *h^2^* = 0.25), then the expectation from drift will fall to 4.25 and 0.15 standard deviates for large and small *N_e_* instead of 8.5 and 3.0. If this is the case, then the observed differentiation is still less than expected from drift under small *N_e_*, but is greater than drift under large *N_e_*.

Drift also depends on population size. The concept of “population” in small body sized animals like these shrews is complex because of metapopulation dynamics; the “populations” that would differentiate by drift could be viewed as the two local populations on either side of the hybrid zone, or they could be viewed as the two race metapopulations as units. We estimated drift based on both views. Because we are considering time scales that are several thousands of years long, probably 8,000 to 10,000 shrew generations, the metapopulation view almost certainly needs to be taken into account. Local population densities of *S. araneus* have been estimated in field studies at 5 to 98 animals per ha, not all of which are reproductive; individual home range sizes range from 0.04 to 0.28 ha [68]. We used *N_e_ = *70 as our local population estimate. Long-term effective population sizes of entire races have been estimated at 68,000 to 74,000 based on mtDNA haplotypes [69]. We used *N_e_ = *70,000 as our upper-end metapopulation estimate. The relevant value for our study most likely lies somewhere in between. The observed phenotypic differences at the M-S zone are smaller than expected from both the large or small estimates of effective population size; those at the N-T zone are smaller than the drift under small *N_e_*, but similar in magnitude to those based on large *N_e_*.

The effect of drift also depends on the interval of time over which it operated. Our estimate uses a 10,000 year interval, which is the approximate duration of Marine Oxygen Isotope Stage 2 (MIS2, 14–29 kya [76]), the period during which the Last Glacial Maximum occurred. The races are hypothesized to have lived allopatrically during this period, if not longer (see discussion below). If we make the conservative assumption that the races were isolated only during the most climatically intense part of MIS2 and that differentiation ceased with population expansion 10 kya, then the duration of allopatry could be as little as 10,000 years. If the time spent in allopatry was longer then the expectation of differentiation due to drift will be larger. The entire period since the last interglacial, includes not only MIS2, but also MIS 3 and 4, a period of 57,000 years (14–71 kya [76]). Thus, the races could easily have been allopatrically separated for more than five times the duration of our estimates, but probably not less.

Relaxing our assumptions suggests that only if heritability is very low, *N_e_* very high, and the time spent in allopatry very short, will the phenotypic differences we observe be large enough to match the expectation of drift. Otherwise, our results suggest that phenotypic differentiation has been lost through gene flow because it is less than expected from drift.

### Estimates of time since secondary contact under a model of free gene flow

The estimates of time since secondary contact based on phenotypic differentiation are much shorter than is realistic, indicating that barriers to gene flow must exist. If there was free gene flow across the hybrid zones, then secondary contact would be no more than 0.8 to 158 years ago based on the width of the phenotypic clines (**Table 2**). These clines have almost certainly been in place longer than that. The N-T hybrid zone has been studied for more than 25 years [77], and the two races themselves have been known from locations near the zone for almost 40 years [78], [79]. The M-S zone has been known in its current position for more than a decade [34]. Indeed, even the time taken to produce this paper is many times longer than the lower estimate of 0.8 years since secondary contact. In fact, it is probable that the races have been in contact along hybrid zones for 10,000 years since the beginning of the Holocene, probably for 6,000 years since the end of the Holocene climatic optimum. *S. araneus* is known from fossil cave faunas in the Altai Mountains prior during MIS2 [80], which document that shrews were living near the current position of the Novosibirsk-Tomsk hybrid zone during the last glacial maximum. The only way that the observed tiny levels of phenotypic differentiation can be maintained is with the protection of lowered gene flow. Our estimates of time since secondary contact are based on a dispersal distance of 1 km per generation based on individual home range size [68], [81]. If the dispersal rate was higher, then the estimated time since secondary contact would become shorter. Nevertheless, for the estimated time since secondary contact to be even 8,000 years ago, the dispersal rate would have to be less than 1 cm per year. We therefore conclude that if gene flow was completely free across these hybrid zones, then phenotypic differentiation would long since have been lost given the current widths of the phenotypic clines and the observed shape differences between the hybridizing races.

### Estimates of selection based on *Q_ST_/F_ST_* ratios

Published levels of genetic differentiation (*F_ST_*) were generally smaller or similar in magnitude to phenotypic levels of shape differentiation (*Q_ST_*). At M-S the shape traits had *Q_ST_* values that ranged from 0.012 (lateral mandible) to 0.021 (skulls), and at N-T they ranged from 0.012 (lateral mandible) to 0.040 (skulls) (**Table 1**). By comparison, Horn et al. [29] found *F_ST_  = *0.011 at M-S and *F_ST_  = *0.027 at N-T based set of microsatellite markers from the same populations we sampled for phenotypic differentiation. These figures yield *Q_ST_*/*F_ST_* ratios ranging from 1.09 to 1.91 at M-S and 0.44 to 1.48 at N-T. Grigoryeva et al. [65] found *F_ST_* = 0.05 at M-S using 11 autosomal microsatellite markers, but at a different location than our phenotypic clines. If this estimate of genetic differentiation is transferrable, then the *Q_ST_*/*F_ST_* ratios would drop to 0.24 to 0.42 at M-S.

*Q_ST_* to *F_ST_* ratios near 1 indicate that both phenotypic and neutral genetic markers have differentiated by the same process, which is normally interpreted as drift because of the neutrality of the genetic markers [61]. If the phenotype is under diversifying selection then the ratio will be greater than 1, but if the phenotype is under stabilizing selection in the two races then the ratio will be less than 1 [61]. Our results therefore suggest the possibility that skull shape has undergone diversifying selection. Invoking diversifying selection to explain differences that were identified above as smaller than expected from drift alone is, however, a contradiction. Mandible shape with its lower ratios is consistent with having either evolved by drift or having been subject to stabilizing selection on both sides of the hybrid zones.

## Discussion

Collectively, our results suggest that the M-S and N-T hybrid zones are acting as partial but incomplete barriers to gene flow. Phenotypic differentiation is statistically significant, the clines in phenotype are similar in location and width to the chromosomal clines, and the differentiation is too great to persist in the face of completely unimpeded gene flow. Nevertheless, the amount of differentiation is less than the amount that would have accumulated by drift (or diversifying selection) during the inferred period of allopatry prior to secondary contact, suggesting that at least some differentiation has been lost subsequently. We will discuss each component of this logic here.

The close match between the position and widths of the phenotypic and chromosomal clines suggest that the occurrence of chromosomal heterozygotes helps to maintain the phenotypic clines. Either the karyotypic incompatibilities are maintaining the cline directly as a barrier to gene flow in their own right, or indirectly if gene flow is impeded by genetic incompatibilities instead of chromosomal ones. If there was no inhibition in gene flow whatsoever, then the phenotypic clines would be wider or non-existent given the inferred age of the hybrid contact zones. However, the phenotypic clines at the N-T and M-S zones are comparatively narrow. Chromosomal clines in *S. araneus* are often tens of kilometers wide [5]. The hybrid zone between the Hermitage and Oxford races in Britain, for example, which involves a CV complex heterozygote but which has an acrocentric peak at its center that favors the production of simple rather than complex heterozygotes, and is on average 25 km wide and at points is up to 40 km wide [5], [55]. The Drnholec-Ulm zone in the Czech Republic, which involves only simple heterozygotes (with maximally two CIII meiotic configurations), has chromosomal clines approximately 40 km wide [5], [82]. Clines in other mammal species are also often tens of kilometers wide [75]. The 2.5 to 6.8 km wide phenotypic zones at the M-S and N-T zones are thus comparatively narrow, which provides the basis for our conclusion that they are similar in width to the metacentric clines. The 24.5 to 34.0 km wide clines in mandibular shape at N-T are more typical of phenotypic clines. For all our traits, the phenotypic clines encompassed the chromosomal cline centers, despite their comparative narrowness, suggesting both that the two types of clines are linked and that phenotypic differentiation is not significantly affected by introgression, which would offset the phenotypic centers in one direction or the other (though this may be the case with mandibular shape at the N-T zone).

The lack of substantial phenotypic differences between the hybridizing races suggests that at least some differentiation has been lost to gene flow since secondary contact. However, the narrow phenotypic clines and the incompatibility between the phenotypic and independent estimates of time since secondary contact suggest that the hybrid zones are indeed barriers to gene flow, even if they are not completely impermeable. Interestingly, *Q_ST_/F_ST_* ratios for skull shape hint that there may have been selection for phenotypic differentiation between the hybridizing races, though whether that selection is acting at present or whether it occurred many millennia ago in glacial refugia is unknown. That evidence is weak, however. If the alternative estimates for *F_ST_* are correct, diversifying selection is unsupported by our data.

The overall picture that emerges from our results is one in which long periods of allopatric separation in glacial refugia were accompanied by phenotypic differentiation due to drift, or perhaps selection. After secondary contact in the post-glacial period, probably at least 10,000 years ago based on the widespread distribution of *S. araneus* fossils during glacial times [80, 83 84], partial gene flow allowed some of this differentiation to be lost. That it was partial is indicated by the sharp clines and the incomplete loss of the differences, which have had more than enough time to dissipate completely if gene flow were unimpeded. The Robertsonian incompatibilities between the hybridizing races seem like the most plausible cause of the reduction in gene flow across the tension zones.

Several cycles of glacial differentiation and interglacial introgression have probably occurred over the longer history of these hybridizing races. While the origin of metacentric races has been hypothesized to be associated with post-glacial expansions since the last glacial maximum (LGM) at 21 kya [85], [86], the fossil record of *S. araneus s.l.* extends back 2.5 to 3.0 million years [84]. Paleophylogeographic evidence indicates that some hybridizing races had last common ancestors that predate the last interglacial 125 kya [87], making it possible that the complex system of karyotypic variation has originated iteratively through the last five, ten, or even twenty glacial-interglacial cycles that have occurred during the species' history. If the intraspecific phylogeographic history of *S. araneus* is hundreds of thousands or millions of years deep, then the species would have passed through many allopatric phases split into glacial refugia. The process of speciation in *S. araneus* may be an iterative one, with “two steps forward” during long allopatric glacial cycles and “one step back” due to introgression during interglacial periods. The Robertsonian variation may not completely prevent gene flow, but the phenotypic data suggest that it inhibits enough of it that not all the differentiation is erased. This possibility deserves further investigation with a combination of paleontological, phenotypic, genetic analysis, and phylogenetic analysis.

## Conclusions

Our phenotypic data show that there is significant differentiation across the hybrid zones, that the phenotypic clines are centered on the same places and have similar widths as chromosomal clines, and that the amount of differentiation across the zones is greater than expected if it were dissipating due to gene flow. While some results suggest that differences may have arisen by weak diversifying selection, the preponderance of evidence suggests that they arose through drift.

Chromosome rearrangements are thus confirmed to be a factor that helps maintain phenotypic differentiation in the Common shrew, despite the gene flow that appears to exist at the hybrid zones. Our data refute the idea that allopatrically evolved differences are being rapidly lost through gene flow across the hybrid zones since secondary contact (e.g., [28]), though there is little doubt that some phenotypic differentiation has been erased by slow gene flow. The sharp phenotypic clines we found in several of the skull and mandible shape would be blurred in only a few years if gene flow was unrestricted. In order for these clines to have been maintained for the thousands of years that they have probably existed, gene flow would have to be near zero at the loci that affect skull and mandible shape, which are expected to be spread across the entire set of autosomes. In Common shrews, chromosomal rearrangements are more than incidental in regard to phenotypes.

## Supporting Information

Table S1 **Detailed information about the localities sampled for this study.**
(DOC)

Table S2 **Landmark descriptions.**
(DOC)
