# Supplementary material for: Phenotypic Variation across Chromosomal Hybrid Zones of the Common Shrew (Sorex araneus) Indicates Reduced Gene Flow
Source: PLoS One. 2013 Jul 10;8(7):e67455. doi: 10.1371/journal.pone.0067455 (PMC3707902; doi:10.1371/journal.pone.0067455)
Supplement: Table S1 — Detailed information about the localities sampled for this study. (DOC) [file pone.0067455.s001.doc]

**Table S1.** Localities. *N* is the number of individuals sampled. Race indicates whether only one race, both races (mixed), or whether hybrids (with or without pure race individuals) were found at the locality. Distance is the number of km from the metacentric hybrid zone center (negative toward the Novosibirsk and Seliger sides). * pure race localities used to estimate means but not clines.

| **Locality** | ***N*** | **Race** | **Latitude (N)** | **Longitude (E)** | **Altitude (m)** | **Distance (km)** |
| --- | --- | --- | --- | --- | --- | --- |
| **Novosibirsk-Tomsk Zone** | | | | | | |
| 1 | 8 | Hybrid | 54.808 | 83.43 | 215 | -0.16 |
| 2 | 2 | Novosibirsk | 54.76 | 83.326 | 208 | -7.40 |
| 4 | 1 | Mixed | 54.751 | 83.29 | 206 | -9.80 |
| 5 | 3 | Mixed | 54.774 | 83.376 | 243 | -3.73 |
| 6 | 3 | Hybrid | 54.806 | 83.434 | 229 | 0.08 |
| 10 | 1 | Novosibirsk | 54.741 | 83.349 | 162 | -7.00 |
| 11 | 10 | Hybrid | 54.795 | 83.423 | 270 | -0.43 |
| 12 | 4 | Tomsk | 54.806 | 83.466 | 281 | 2.20 |
| 13 | 3 | Hybrid | 54.806 | 83.436 | 241 | 0.25 |
| 17 | 18 | Hybrid | 54.809 | 83.453 | 274 | 1.28 |
| 19 | 1 | Mixed | 54.806 | 83.44 | 251 | 0.48 |
| 22 | 1 | Hybrid | 54.806 | 83.442 | 250 | 0.61 |
| 23 | 23 | Hybrid | 54.792 | 83.426 | 270 | -0.44 |
| 24 | 2 | Mixed | 54.807 | 83.444 | 255 | 0.73 |
| 27 | 7 | Hybrid | 54.788 | 83.438 | 264 | 0.39 |
| 30 | 12 | Hybrid | 54.811 | 83.456 | 272 | 1.44 |
| 32 | 14 | Hybrid | 54.782 | 83.445 | 254 | 0.89 |
| 33 | 1 | Novosibirsk | 54.773 | 83.305 | 141 | -8.70 |
| 34 | 1 | Novosibirsk | 54.79 | 83.4 | 235 | -2.05 |
| 35 | 5 | Mixed | 54.763 | 83.389 | 244 | -3.27 |
| aje17-25* | 5 | Tomsk | 54.757 | 87.027 | 150 |  |
| akad17-25* | 5 | Novosibirsk | 54.82 | 83.118 | 180 |  |
| **Moscow-Seliger Zone** | | | | | | |
| 1 | 5 | Hybrid | 32.661 | 57.277 | 215 | -2.55 |
| 2 | 10 | Seliger | 32.674 | 57.275 | 219 | -2.33 |
| 3 | 8 | Hybrid | 32.668 | 57.276 | 217 | -2.43 |
| 4 | 3 | Seliger | 32.669 | 57.26 | 235 | -0.61 |
| 5 | 5 | Hybrid | 32.661 | 57.262 | 232 | -0.98 |
| 6 | 12 | Hybrid | 32.674 | 57.256 | 237 | -0.3 |
| 7 | 12 | Hybrid | 32.669 | 57.256 | 236 | -0.15 |
| 8 | 13 | Hybrid | 32.665 | 57.255 | 234 | -0.22 |
| 9 | 6 | Hybrid | 32.662 | 57.255 | 233 | -0.35 |
| 10 | 5 | Hybrid | 32.659 | 57.254 | 236 | -0.49 |
| 11 | 13 | Hybrid | 32.66 | 57.253 | 236 | -0.37 |
| 12 | 14 | Hybrid | 32.666 | 57.253 | 236 | -0.13 |
| 13 | 8 | Hybrid | 32.67 | 57.254 | 233 | 0.03 |
| 14 | 7 | Hybrid | 32.675 | 57.254 | 232 | -0.13 |
| 16 | 6 | Moscow | 32.658 | 57.236 | 232 | 0.84 |
| 17 | 7 | Moscow | 32.665 | 57.236 | 231 | 1.01 |
| 18 | 10 | Hybrid | 32.671 | 57.235 | 229 | 1.17 |
| A | 5 | Seliger | 32.574 | 57.307 | 206 | -8.03 |
| B | 3 | Seliger | 32.952 | 57.376 | 224 | -10.03 |
