# Supplementary material for: Phenotypic Variation across Chromosomal Hybrid Zones of the Common Shrew (Sorex araneus) Indicates Reduced Gene Flow
Source: PLoS One. 2013 Jul 10;8(7):e67455. doi: 10.1371/journal.pone.0067455 (PMC3707902; doi:10.1371/journal.pone.0067455)
Supplement: Table S2 — Landmark descriptions. (DOC) [file pone.0067455.s002.doc]

**Table S2**. Landmark descriptions.

*Cranium* (25 landmarks):

1. midline of the posterior margin of the foramen magnum.

2. midline of the anterior margin of the foramen magnum.

3. midline of the posterior margin of the palate.

4. midline of the sutures between the palatine and maxilla.

5. anterior point of the midline suture between the premaxillae.

6-11, 12, 14, 16. buccal side of the point at which adjacent teeth meet, from the incisor through the third molar.

13, 15, 17. palatal tip of the protocone of the first through third molars.

18. intersection between the lateral margin of the pterygoid plates and the posterior margin of the palate.

19. intersection between the medial margin of the pterygoid plates and the posterior margin of the palate.

20. anterior margin of the glenoid fossa.

21. mastoid process.

22. lateral end of the posterior margin of the occipital condyle.

23. center of the posterior lacerate foramen.

24. center of the anterior margin of the occipital condyle.

25. medial end of the posterior margin of the occipital condyle.

*Medial mandible* (23 landmarks).

1. inferior end of the alveolus of the incisor.

2-7. posterior ends of the alveoli of the mandibular teeth.

8. anterior margin of the superior crest of the coronoid process.

9. posterior margin of the superior crest of the coronoid process.

10. anterior end of the inferior margin of the opening to the fossa temporalis.

11. posterior end of the inferior margin of the opening to the fossa temporalis.

12. apex of the fossa temporalis.

13. inferior point of the saddle between the condylar and coronoid processes.

14. medial end of the superior facet of the mandibular condyle.

15. lateral end of the superior facet of the condyle.

16. medial end of the inferior facet of the condyle.

17. superior side of the junction of the angular process to the body of the mandible.

18. posterior margin of the mandibular foramen.

19. anterior margin of the mandibular foramen.

20. inferior side of the junction of the angular process to the body of the mandible.

21. inferiormost point of the posterior convex saddle of the body of the mandible.

22. superiormost point of the concave saddle of the body of the mandible.

23. inferiormost point of the anterior convex saddle of the body of the mandible.

*Lateral mandible* (17 landmarks).

1. inferior margin of the alveolus of the incisor.

2-7. posterior ends of the alveoli of the mandibular teeth.

8. anterior margin of the superior crest of the coronoid process.

9. posterior margin of the superior crest of the coronoid process.

10. inferior point of the saddle between the condylar and coronoid processes.

11. lateral end of the superior facet of the condyle.

12. medial end of the inferior facet of the condyle.

13. superior side of the junction of the angular process to the body of the mandible.

14. superior side of the junction of the angular process to the body of the mandible.

15. inferiormost point of the posterior convex saddle of the body of the mandible.

16. superiormost point of the concave saddle of the body of the mandible.

17. mental foramen.
